# Impaired immunogenicity after vaccination for SARS-CoV-2 in patients with gastrointestinal cancer: does tumor entity matter?

**DOI:** 10.21037/jgo-22-1065

**Published:** 2023-06-26

**Authors:** Malte Benedikt Monin, Jens Gabriel Gorny, Moritz Berger, Leona I. Baier, Taotao Zhou, Robert Mahn, Farsaneh Sadeghlar, Christian Möhring, Christoph Boesecke, Kahtrin van Bremen, Gereon J. Rieke, Stefan Schlabe, Stefan Breitschwerdt, Milka Marinova, Ingo G. H. Schmidt-Wolf, Christian P. Strassburg, Anna-Maria Eis-Hübinger, Maria A. Gonzalez-Carmona

**Affiliations:** 1 Department of Internal Medicine I, University Hospital Bonn, Bonn, Germany;; 2 German Centre for Infection Research (DZIF), Partner-site Cologne-Bonn, Bonn, Germany;; 3 Institute for Medical Biometry, Informatics and Epidemiology, Bonn University Hospital, Bonn, Germany;; 4 Department of Nuclear Medicine, University Hospital Bonn, Bonn, Germany;; 5 Department of Integrated Oncology, Center for Integrated Oncology (CIO), University Hospital Bonn, Bonn, Germany;; 6 Institute of Virology, Bonn University Hospital, Bonn, Germany

**Keywords:** Vaccination for SARS-CoV-2, gastrointestinal cancer, SARS-CoV-2 immunogenicity, SARS-CoV-2 surrogate neutralization antibodies, vaccination failure

## Abstract

**Background:**

SARS-CoV-2 immunogenicity in patients with gastrointestinal cancer (GI cancer) following second and third vaccination was analyzed.

**Methods:**

A total of 125 patients under active anticancer therapy or in follow-up care were included in this prospective study. Seroprevalence of SARS-CoV-2 anti-spike and surrogate neutralization antibodies (NABs) was measured.

**Results:**

Four weeks after second vaccination, adequate titers of SARS-CoV-2 anti-spike immunoglobulin G (IgG) [≥282.0 binding antibody units (BAU)/mL] were found in 62.2% of patients under treatment versus 96.3% of patients in follow-up care (P<0.01). Sufficient titers of SARS-CoV-2 surrogate NAB (≥85.0%) were found in 32.7% of patients under treatment versus 70.6% in follow-up care (P<0.01). Titers of SARS-CoV-2 anti-spike IgG were especially low in patients with colorectal cancer (CRC). For SARS-CoV-2 surrogate NAB, patients with hepatocellular carcinoma (HCC) and with pancreaticobiliary cancer showed the lowest titers (P<0.01). SARS-CoV-2 anti-spike IgG and SARS-CoV-2 surrogate NAB were associated with a correlation coefficient of 0.93. Reaching a titer of SARS-CoV-2 anti-spike IgG ≥482.0 BAU/mL, protective levels of SARS-CoV-2 surrogate NAB (≥85.0%) could be assumed. Following booster vaccination, all patients reached effective antibody titers.

**Conclusions:**

Patients with active GI cancer showed impaired immunogenicity after second SARS-CoV-2 vaccination which was overcome by booster vaccination. Our findings were tumor-related and pronounced in patients with CRC and HCC. Waning immunity over time and antibody escape phenomena by variant of concern Omicron must be considered in these especially vulnerable patients.

**Highlight box**

**Key findings**

• Impaired immunogenicity after second SARS-CoV-2 vaccination was overcome by booster vaccination.

**What is known and what is new?**

• Impaired immunogenicity in patients with several solid cancer types has already been described. Yet, differentiated data for patients with gastrointestinal cancer are missing.

• Patients with gastrointestinal cancer showed impaired immune responses follwing vaccination for SARS-CoV-2.

**What is the implication, and what should change now?**

• Booster vaccinations for SARS-CoV-2 should be recommended to patients with gastrointestinal cancer.

• In patients with gastrointestinal cancer being infected with SARS-CoV-2, antiviral therapy should be taken into consideration tp prevent severe COVID-19.

## Introduction

SARS-CoV-2 infected patients with cancer, especially under active cancer treatment, are facing higher rates of morbidity and mortality compared to healthy people (1,2). Both, the American Society for Clinical Oncology (ASCO) and the European Society for Medical Oncology (ESMO) recommended to prioritize patients with cancer in vaccination campaigns (3,4). However, actively treated patients with cancer were excluded from trials analyzing efficacy of SARS-CoV-2 vaccines (5-9).

First data concerning immunogenicity to SARS-CoV-2 revealed reduced response rates to vaccination in patients with cancer. However, most of the studies focused on patients with hematological cancer or included different types of solid cancer without differentiating between tumor types (10-19). For the time being, differentiated data on patients with gastrointestinal (GI) cancer are sparse (20). Antibody titers were compared to people in control groups without any history of cancer who were considerably healthier and younger. Finally, only a small number of studies referred to neutralization antibodies (NABs) as being decisive for immune protection from symptomatic SARS-CoV-2 (12-19,21-23). No titer could be defined as being linked to protection from severe coronavirus disease 2019 (COVID-19). Especially liver dysfunction due to primary hepatobiliary tumors or secondary hepatic metastases from GI tumors as well as due to underlying liver steatosis, fibrosis and/ or cirrhosis is associated with immunodeficiency resulting in impaired immune responses to well-known vaccines (24-28). Taking all these information into consideration, a more detailed analysis of immunogenicity in patients with GI tumors is necessary for making recommendations concerning additional booster vaccinations in these patients.

Thus, in the present study, we provide novel data on response rates to basic and booster vaccination for SARS-CoV-2 in patients with GI cancer under anticancer treatment but also in follow-up care, offering robust evidence for recommendations on antibody assessment, individual booster vaccinations as well as on passive immunization and/or antiviral therapy in individual patients with GI cancer. We present this article in accordance with the STROBE reporting checklist (available at https://jgo.amegroups.com/article/view/10.21037/jgo-22-1065/rc).

## Methods

### Study design

This is a prospective, observational, longitudinal study on the efficacy of vaccination for SARS-CoV-2 in patients with GI cancer treated at the Department of Internal Medicine I, gastroenterology oncology section at the University Hospital Bonn between January 2021 and April 2022. We focused on humoral immunogenicity for SARS-CoV-2 in patients with GI cancer.

Total antibody titers as well as titers of surrogate NAB were considered and titers probably linked to protection from severe COVID-19 were defined for our cohort of patients. Blood samples were drawn to analyze seroprevalence of SARS-CoV-2 anti-spike antibodies 4, 12 and 24 weeks after second vaccination for SARS-CoV-2. After 12 weeks, SARS-CoV-2 surrogate NAB were additionally measured. Four and 12 weeks after booster vaccination, we again assessed antibody titers. During the study period, we screened for infections with SARS-CoV-2. The study was performed in accordance with the Declaration of Helsinki (as revised in 2013) and approved by the institutional review board of the Medical Faculty of the University of Bonn (No. 341/17 and 023/22). Written informed consent was obtained from all participating patients.

### Patient characteristics and eligibility criteria

In descending order, patients with pancreaticobiliary neoplasms [PBN: cholangiocarcinoma (CCC), papillary carcinoma, gallbladder cancer (GBC), pancreas cancer], hepatocellular cancer (HCC), colorectal cancer (CRC), neuroendocrine tumors (NETs), upper GI tract cancer [esophageal, gastro-esophageal junction (GEJC), gastric cancer], cancer of unknown primary (CUP) with most likely GI origin as well as gastrointestinal stromal tumors (GISTs) were included. Any kind of ongoing oncological treatment (chemotherapy, targeted therapy, immune checkpoint inhibition, local therapy, or combination of different therapeutic strategies) was eligible for inclusion. Patients being off treatment but having undergone oncological treatment within the past 12 months were also included in this group. Patients in follow-up care being at least 12 months without detectable tumor and having been off treatment >12 months were included as control group. These patients share comparable risk factors, both for cancer pathogenesis as well as for severe COVID-19 and for impaired immune responses to vaccinations. Relevant clinical information, especially regarding COVID-19 infections, patients’ performance [Eastern Cooperative Oncology Group (ECOG) score] and disease status (local, tumor recurrence and metastatic), were obtained from standardized medical records. Increased immunosuppression was suspected in patients with a medical history of autoimmune disease or organ transplantation with concomitant therapy with corticosteroids, methotrexate or calcineurin inhibitors as well as in patients with uncontrolled human immunodeficiency virus (HIV) infections (*Table 1*).

**Table 1:** Patient baseline characteristics

Patients	Under treatment (N=85)	Off treatment >1 year (N=40)	P
Age (years), median [IQR]	67 [61–75]	68 [60–72]	0.47
Sex, n (%)			0.12
Women	31 (36.5)	21 (52.5)	
Men	54 (63.5)	19 (47.5)	
Tumor types, n (%)			0.02
PBN	21 (24.5)	5 (12.5)	
CCC	9 (10.5)	1 (2.5)	
GBC	1 (1.0)	1 (2.5)	
Pancreas cancer	11 (13.0)	3 (7.5)	
HCC	20 (23.5)	7 (17.5)	
CRC	17 (20.0)	13 (32.5)	
NET	13 (15.0)	1 (2.5)	
GEJC	9 (11.0)	11 (27.5)	
Gastric cancer	4 (5.0)	6 (15.0)	
Oesophageal cancer	5 (6.0)	5 (12.5)	
Other	5 (6.0)	3 (7.5)	
CUP	3 (4.0)	2 (5.0)	
GIST	2 (2.0)	1 (2.5)	
Disease status, n (%)			<0.01
Local	32 (37.5)	34 (85.0)	
Tumor recurrence	10 (12.0)	0 (0.0)	
Metastatic	43 (50.5)	6 (15.0)	
Intention of treatment, n (%)			
Neoadjuvant	13 (15.0)		
Adjuvant	12 (14.0)		
Palliative	60 (71.0)		
Type of treatment, n (%)			
Chemotherapy^a^	32 (38.0)		
Targeted therapy^b^	14 (16.0)		
Immune checkpoint inhibition	4 (5.0)		
Local therapy^c^	12 (14.0)		
Combined therapy^d^	23 (27.0)		
Additional immunosuppression^e^	14 (16.5)	2 (5.0)	0.09
Performance status (ECOG score), n (%)			<0.01
0	36 (77.5)	31 (43.4)	
1	39 (22.5)	9 (47.0)	
2	8 (9.6)	0 (0.0)	
Risk factors for severe COVID-19, n (%)			
Age >65 years	66 (78.0)	29 (72.5)	0.65
BMI >30 kg/m^2^	6 (7.0)	1 (2.5)	0.43
History of smoking	21 (25.0)	13 (32.5)	0.39
Hypertension	49 (58.0)	19 (47.5)	0.34
Chronic respiratory disease	6 (7.0)	8 (20.0)	0.06
Cardiovascular disease	13 (15.0)	9 (22.5)	0.33
Chronic kidney disease	7 (8.0)	1 (2.5)	0.43
Liver disease	23 (27.0)	5 (12.5)	0.11
Neurological disorder	5 (6.0)	2 (5.0)	1.00
Organ transplant	3 (3.5)	0 (0.0)	0.55
Autoimmune disease	5 (6.0)	1 (2.5)	0.66
Diabetes mellitus	21 (25.0)	5 (12.5)	0.16
History of SARS-CoV-2 infection prior to vaccination (self-report, serology or PCR testing), n (%)	4 (5.0)	1 (2.5)	1.0
History of SARS-CoV-2 infection after second vaccination, (self-report, serology or PCR testing), n (%)	4 (5.0)	1 (2.5)	1.0
History of SARS-CoV-2 infection after booster vaccination, (self-report, serology or PCR testing), n (%)	3 (4.0)	1 (2.5)	1.0
Vaccine, n (%)			0.86
BNT162b2 (Pfizer & BioNTech)	73 (86.0)	33 (82.5)	
AZD1222 (AstraZeneca)	6 (7.0)	3 (7.5)	
mRNA-1273 (Moderna)	2 (2.0)	2 (5.0)	
AZD1222 + BNT162b2/mRNA-1273	3 (4.0)	2 (5.0)	
Ad26.COV2.s (Johnson & Johnson, Janssen)	1 (1.0)	0 (0.0)	
Patients with SARS-CoV-2 anti-spike IgG ≥282.0 BAU/mL (effective titer)			
4 weeks after second vaccination	62.2% (46/74)	96.3% (26/27)	<0.01
12 weeks after second vaccination	39.3% (22/56)	60.0% (21/35)	0.09
4 weeks after booster vaccination	100.0% (27/27)	100.0% (17/17)	
Patients with SARS-CoV-2 surrogate NAB ≥85.0% (effective titer)			
12 weeks after vaccination	32.7% (17/52)	70.6% (24/34)	<0.01
4 weeks after booster vaccination	100.0% (22/22)	100.0% (17/17)	

Baseline characteristics were compared between treatment and control group using Student’s *t*-test for continuous variables and Fisher’s exact tests for the categorical variables. ^a^, mostly with combinations of 5-fluorouracil, platinum derivates, gemcitabine, taxane or irinotecan; ^b^, including multikinase inhibitors such as sorafenib, lenvatinib, cabozantinib, EGFR (epidermal growth factor receptor) antibodies or VEGF (vessel endothelial growth factor) antibodies; ^c^, including transarterial embolization, radioembolization or endobiliary/transcutaneous radiofrequency ablation and photodynamic therapy; ^d^, chemotherapy + targeted therapy or chemotherapy + local therapy; ^e^, co-medication with corticosteroids, methotrexate or calcineurin inhibitor or HIV-infection. IQR, interquartile range; PBN, pancreaticobiliary neoplasm; CCC, cholangiocellular cancer; GBC, gallbladder cancer; HCC, hepatocellular cancer; CRC, colorectal cancer; NET, neuroendocrine tumor; GEJC, gastro-oesophageal-junction cancer; CUP, carcinoma of unknown primary; GIST, gastrointestinal stromal tumor; ECOG, Eastern Cooperative Oncology Group; COVID-19, coronavirus disease 2019; BMI, body mass index; PCR, polymerase chain reaction; BAU, binding antibody units; NAB, neutralization antibody.

All vaccines for SARS-CoV-2 approved in Germany could be administered (BNT162b2 by Pfizer BioNTech, AZD1222 by AstraZeneca, mRNA-1273 by Moderna, Ad26.COV2.S by Johnson & Johnson Janssen).

### Assessment of SARS-CoV-2 antibodies

SARS-CoV-2 immunoglobulin G (IgG) II Quant chemiluminescent microparticle immunoassay (Abbott Laboratories, Chicago, USA) was used to quantify IgG antibodies against SARS-CoV-2 spike receptor-binding domain (SARS-CoV-2 anti-spike IgG). Values ≥7.1 binding antibody units per milliliter (BAU/mL) were evaluated as positive though no threshold for protection was defined. In our study, mean estimated SARS-CoV-2 anti-spike IgG was 282.0 BAU/mL in patients in follow-up care 12 weeks after second vaccination. Titers ≥282.0 BAU/mL were thus regarded as being associated with most likely protection from severe COVID-19 in our cohort as only one patient of this group had mild COVID-19 after two vaccinations.

To identify the portion of SARS-CoV-2 NABs in relation to all antibodies [%], a blocking enzyme-linked immunosorbent assay (ELISA) detection tool (cPass^TM^ SARS-CoV-2 Neutralization Antibody Detection Kit; GenScript, New Jersey, USA) was used. This test detects functional virus neutralization strongly correlating with live-cell neutralization (29,30). We thus report on SARS-CoV-2 surrogate NAB as we did not explicitly measure live-cell neutralization. Reaching values ≥30% was defined as positive with no threshold for protection. The mean estimated SARS-CoV-2 surrogate NAB titer 12 weeks after second vaccination was 84.81% in patients in follow-up care in our cohort. Therefore, titers ≥85.0% were again regarded as probably equivalent to protection from severe COVID-19.

Elecsys anti-SARS-CoV-2 chemiluminescent immunoassay (Roche) was used for qualitative detection of SARS-CoV-2 anti-nucleocapsid IgG in patients having undergone an infection with SARS-CoV-2 prior to or despite vaccination.

### Statistical analysis

Statistical analysis was carried out using R version 4.1.1 (R Core Team 2021: R: A Language and Environment for Statistical Computing, R Foundation for Statistical Computing, Vienna, Austria). Descriptive analyses included the calculation of mean and interquartile range for continuous variables and frequencies (absolute and relative) for categorical variables. Association between levels of SARS-CoV-2 anti-spike IgG and SARS-CoV-2 surrogate NAB was analyzed using Spearman’s rank correlation coefficient. Proportions of patients with effective SARS-CoV-2 anti-spike IgG and/or effective SARS-CoV-2 surrogate NAB were compared by chi-square tests. Difference in (log_10_ transformed) levels of SARS-CoV-2 anti-spike IgG in patients with and without effective SARS-CoV-2 surrogate NAB was examined by* t*-test.

Univariate linear mixed regression models were used to compare (log_10_ transformed) levels of SARS-CoV-2 anti-spike IgG (including measurements at 4 and 12 weeks) with respect to treatment status, tumor type, disease status, intention of treatment and type of treatment. Analogously, univariate linear regression models were applied to compare levels of SARS-CoV-2 surrogate NAB (measurements at 12 weeks) with respect to the five discussed influencing factors. Furthermore, multivariable regression analysis was performed to examine a possible effect of age, sex, additional immunosuppression and history of SARS CoV-2 infection on SARS-CoV-2 antibodies, respectively.

## Results

### Patient baseline characteristics

A total of 125 patients with GI cancer were included, of whom 68.0% (N=85) were under treatment and 32.0% (N=40) in follow-up care. In patients under treatment, the most common tumor type was of the pancreato-hepatobiliary type (PBN with 24.5% and HCC with 23.5%) followed by CRC (20.0%). Related to the prognosis of the different GI cancers, patients in follow-up care mainly suffered from CRC (32.5%) followed by GEJC (27.5%) leading to a different distribution of tumor types between the groups (P=0.02). As expected, patients under treatment had more frequently a metastatic disease status than patients in the control group (50.5% *vs.* 15.0%; P<0.01). Patients in both groups had a low ECOG score between 0 and 2. No further significant differences between the groups were observed (*Table 1*).

### Seropositivity for SARS-CoV-2 anti-spike IgG

**Figure 1:**
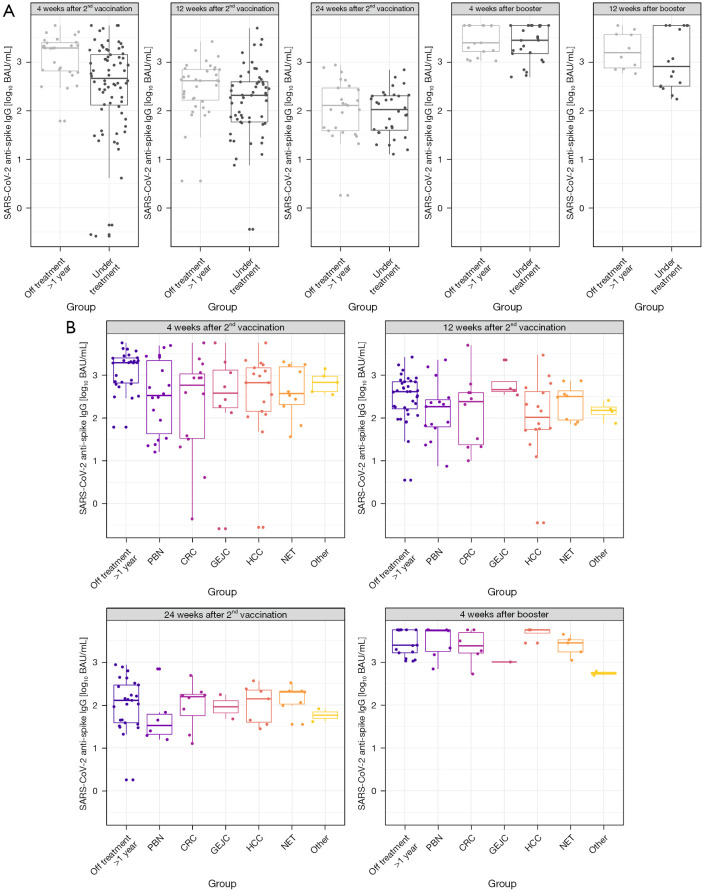
SARS-CoV-2 anti-spike IgG titers. Log_10_ SARS-CoV-2 anti-spike IgG titer of patients off treatment >1 year and patients under treatment at week 4, 12, 24 following second vaccination and at week 4 and week 12 after booster vaccination (A), impact of tumor types (B). Length of box represents the interquartile range, horizontal line shows the mean log_10_ SARS-CoV-2 anti-spike IgG titer, the whiskers denote the area which contains 95% of the data. PBN = pancreas cancer, cholangiocellular cancer, gallbladder cancer; GEJC = gastric cancer, oesophageal cancer; Other = gastrointestinal stromal tumor, carcinoma of unknown primary, combined therapy = chemotherapy + targeted therapy or chemotherapy + local therapy. IgG, immunoglobulin G; BAU, binding antibody units; PBN, pancreaticobiliary neoplasms; CRC, colorectal cancer; GEJC, gastro-oesophageal-junction cancer; HCC, hepatocellular cancer; NET, neuroendocrine tumor.

Mean antibody concentrations of SARS-CoV-2 anti-spike IgG were significantly lower in patients of the treatment group [2.47 log_10_ BAU/mL; 95% confidence interval (CI): 2.29 to 2.64; P<0.01] than in patients in follow-up care (3.06 log_10_ BAU/mL; 95% CI: 2.79 to 3.32) four weeks after second vaccination (*Table 2* and *Figure 1*). SARS-CoV-2 anti-spike IgG titers already decreased at week 12 after second vaccination in patients under active treatment (2.11 log_10_ BAU/mL; 95% CI: 1.92 to 2.29; P=0.08), but also in patients in follow-up care (2.45 log_10_ BAU/mL; 95% CI: 2.20 to 2.71; P<0.01). The differences in mean antibody titers between treatment and control group decreased at week 12 compared to week four after second vaccination, independent of tumor type, disease status, type of treatment or intention of treatment, but was especially pronounced in patients with CRC (2.18 log_10_ BAU/mL; 95% CI: 1.74 to 2.61; P=0.01) (*Table 2* and *Figure 1*).

**Table 2:** Titers of SARS-CoV-2 anti-spike IgG and SARS-CoV-2 surrogate NAB following vaccination

	SARS-CoV-2 anti-spike IgG								SARS-CoV-2 surrogate NAB		
	4 weeks				12 weeks				12 weeks		
	Estimate (log_10_ BAU/mL)	95% CI	P		Estimate (log_10_ BAU/mL)	95% CI	P		Estimate (%)	95% CI	P
Treatment											
Off treatment >1 year (Ref.)	3.06	2.79–3.32			2.45	2.20–2.71	<0.01		84.81	74.80–94.83	
Under treatment	2.47	2.29–2.64	<0.01		2.11	1.92–2.29	0.08		61.38	53.29–69.48	<0.01
Tumor type											
Off treatment >1 year	3.06	2.79–3.32			2.45	2.20–2.71	<0.01		84.81	74.80–94.83	
PBN^a^	2.50	2.14–2.85	0.02		2.16	1.78–2.54	0.12		56.63	40.99–72.27	<0.01
CRC	2.18	1.78–2.57	<0.01		2.18	1.74–2.61	0.01		60.36	39.67–81.05	0.04
GEJC^b^	2.44	1.89–2.89	0.04		2.18	1.53–2.84	0.26		88.30	59.04–117.56	0.83
HCC	2.52	2.15–2.88	0.02		2.05	1.67–2.43	0.47		55.31	40.20–70.42	<0.01
NET	2.60	2.15–3.06	0.10		2.07	1.59–2.55	0.75		65.70	45.01–86.39	0.11
Other^c^	2.82	2.11–3.53	0.55		2.12	1.38–2.86	0.73		69.30	35.51–103.09	0.39
Disease status											
Local (Ref.)	2.80	2.59–3.01			2.30	2.09–2.51			74.81	65.89–83.74	
Metastatic	2.58	2.35–2.81	0.16		2.18	1.93–2.42	0.47		68.13	57.02–79.24	0.36
Tumor recurrence	1.85	1.30–2.39	<0.01		1.86	1.32–2.41	0.05		53.21	29.83–76.60	0.09
Type of treatment											
Off treatment >1 year (Ref.)	3.06	2.79–3.32			2.45	2.20–2.71	<0.01		84.81	74.80–94.83	
Chemotherapy	2.42	2.14–2.71	<0.01		2.02	1.71–2.33	0.25		60.22	47.03–73.40	<0.01
Immunotherapy	2.77	1.99–3.56	0.51		2.35	1.42–3.29	0.64		95.65	55.01–136.29	0.61
Targeted therapy	2.63	2.20–3.07	0.11		2.34	1.88–2.79	0.17		76.88	56.56–97.19	0.49
Local therapy	2.54	2.05–3.03	0.07		1.84	1.32–2.37	0.74		49.33	27.61–71.05	<0.01
Combined therapy^d^	2.37	2.03–2.70	<0.01		2.13	1.78–2.48	0.04		56.02	41.65–70.39	<0.01
Intention of treatment											
Off treatment >1 year (Ref.)	3.06	2.79–3.32			2.45	2.20–2.71	<0.01		84.81	74.80–94.83	
Neoadjuvant	2.00	1.55–2.45	<0.01		1.72	1.25–2.19	0.15		58.37	38.72–78.01	0.02
Adjuvant	2.41	1.95–2.88	0.02		2.38	1.87–2.89	0.03		54.60	28.24–80.96	0.04
Palliative	2.58	2.37–2.78	0.01		2.15	1.93–2.37	0.22		62.99	53.43–72.55	<0.01

Shown are the results of linear mixed model analysis for the SARS-CoV-2 anti-spike IgG and linear model analysis for the SARS-CoV-2 surrogate NAB. Results are reported by mean estimates, 95% confidence intervals and associated P values. ^a^, pancreas cancer, cholangiocellular cancer, gallbladder cancer; ^b^, gastric cancer, oesophageal cancer; ^c^, gastrointestinal stromal tumor, carcinoma of unknown primary; ^d^, chemotherapy + targeted therapy or chemotherapy + local therapy. IgG, immunoglobulin G; NAB, neutralization antibody; Ref., reference; PBN, pancreaticobiliary neoplasm; CRC, colorectal cancer; GEJC, gastro-oesophageal-junction cancer; HCC, hepatocellular cancer; NET, neuroendocrine tumor; BAU, binding antibody units.

In self-report and/or polymerase chain reaction (PCR) testing, 1 infection with SARS-CoV-2 in patients in follow-up care (2.5%) versus 4 infections in patients in the treatment group (5.0%) were documented after two vaccinations (*Table 1*). Effective titers of SARS-CoV-2 anti-spike IgG probably linked to protection from severe COVID-19 were thus defined by values ≥2.45 log_10 _BAU/mL, i.e., ≥282.0 BAU/mL, the mean estimated titer in patients in follow-up care 12 weeks after second vaccination. A total of 62.2% (N=46/74) of patients reached effective titers despite active GI cancer and anticancer treatment 4 weeks after second vaccination. This response rate was significantly reduced compared to patients in follow-up care, who showed effective titers in 96.3% of patients (N=26/27; P<0.01) (*Table 1*). Compared to the titers after 4 weeks, 12 weeks after second vaccination adequate response rates concerning SARS-CoV-2 anti-spike IgG (≥282.0 BAU/mL) were significantly reduced to 39.3% (N=22/56; P=0.02) of patients under treatment versus to 60.0% (N=21/35; P<0.01) of patients in follow-up care. The difference between the groups decreased and was non-significant at this point of time (P=0.09) (*Table 1*).

### Seropositivity for SARS-CoV-2 surrogate NABs

**Figure 2:**
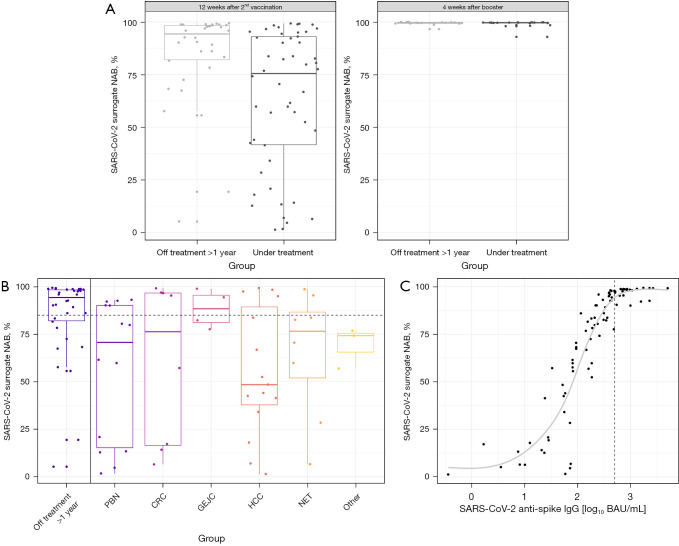
SARS-CoV-2 surrogate NAB titers and association between log_10_ SARS-CoV-2 anti-spike IgG titers and SARS-CoV-2 surrogate NAB titers. SARS-CoV-2 surrogate NAB titers of patients off treatment >1 year and patients under treatment at week 12 following second vaccination and week four after booster vaccination (A), impact of tumor types (B), association between log_10_ SARS-CoV-2 anti-spike IgG titer and SARS-CoV-2 surrogate NAB titer with coefficient of 0.93 (C). Length of box represents the interquartile range, horizontal line shows the mean SARS-CoV-2 surrogate NAB titer, the whiskers denote the area which contains 95% of the data; horizontal dashed line corresponds to a proportion of 85.0% SARS-CoV-2 surrogate NAB; vertical dashed line corresponds to the log_10_ equivalent of 482.0 BAU/mL (corresponding to 2.68 log_10_ BAU/mL) being linked to effective titers of SARS-CoV-2 surrogate NAB (≥85.0%). PBN = pancreas cancer, cholangiocellular cancer, gallbladder cancer; GEJC = gastric cancer, oesophageal cancer; Other = gastrointestinal stromal tumor, carcinoma of unknown primary, combined therapy = chemotherapy + targeted therapy or chemotherapy + local therapy. NAB, neutralization antibody; PBN, pancreaticobiliary neoplasms; CRC, colorectal cancer; GEJC, gastro-oesophageal-junction cancer; HCC, hepatocellular cancer; NET, neuroendocrine tumor; IgG, immunoglobulin G.

Additionally, we focused on SARS-CoV-2 surrogate NAB 12 weeks after second vaccination. Mean titers differed significantly between patients under treatment (61.38%; 95% CI: 53.29% to 69.48%; P*<*0.01) and patients in follow-up care (84.81%; 95% CI: 74.80% to 94.83%). Impairment was most obvious in patients with HCC (55.31%; 95% CI: 40.20% to 70.42%; P<0.01) and PBN (56.63%; 95% CI: 40.99% to 72.27%; P<0.01). Patients under immune checkpoint inhibition had non-significantly higher SARS-CoV-2 surrogate NAB titers (95.65%; 95% CI: 55.01% to 136.29%; P=0.61) (*Table 2* and *Figure 2*).

Again, the mean estimated titer of patients in follow-up care 12 weeks after second vaccination (≥85.0%) was defined as effective. Titers of SARS-CoV-2 surrogate NAB ≥85.0% were found in 32.7% (N=17/52) of patients under treatment versus 70.6% (N=24/34) in patients of the control group, marking a significant difference (P<0.01) (*Table 1*).

### Impact of immunosuppression, age, sex, and overcome COVID-19 on SARS-CoV-2 immunogenicity after second vaccination

In a multivariate analysis, we focused on co-factors potentially influencing immunogenicity after vaccination for SARS-CoV-2.

**Figure 3:**
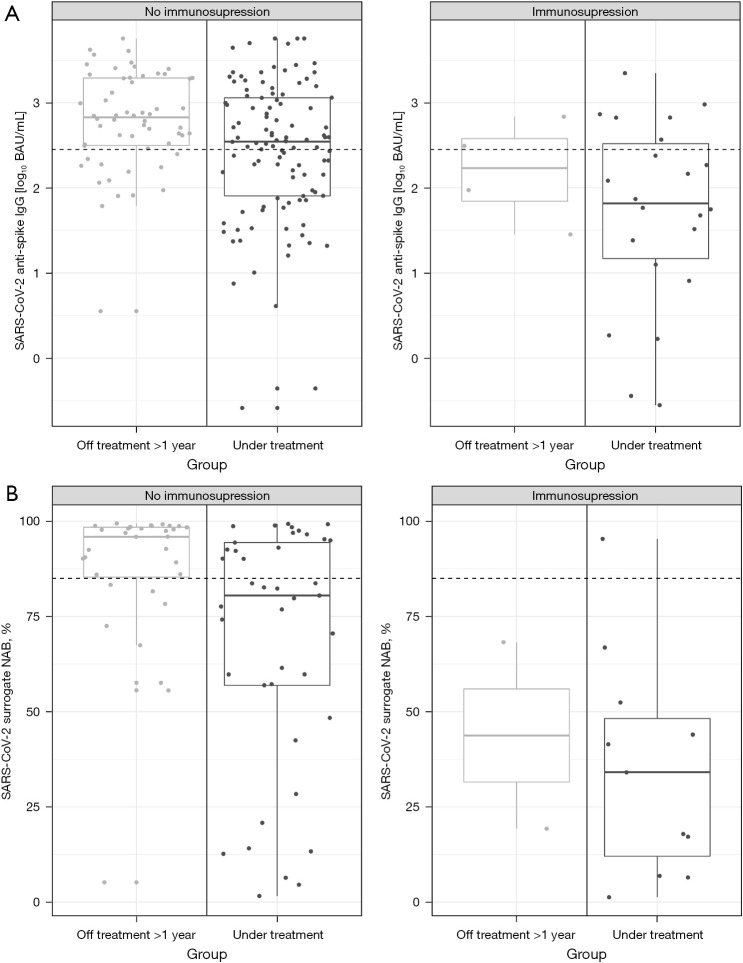
Effect of immunosuppression on antibody titers. Log_10_ SARS-CoV-2 anti-spike IgG titer (A) and SARS-CoV-2 surrogate NAB titer (B) of patients off treatment >1 year and patients under treatment according to the effect of immunosuppression (co-medication with corticosteroids, methotrexate or calcineurin inhibitor, HIV infection, or chronic hepatitis B/C-infection), data 12 weeks after vaccination. Length of box represents the interquartile range, horizontal line shows the mean log_10_ SARS-CoV-2 anti-spike IgG titer (B)/SARS-CoV-2 surrogate NAB titer (A), the whiskers denote the area which contains 95% of the data; dashed line correspond to the log_10_ equivalent of 282.0 BAU/mL (2.45 log_10_ BAU/mL) (A) or 85.0% (B). IgG, immunoglobulin G; BAU, binding antibody units; NAB, neutralization antibody; HIV, human immunodeficiency virus.

In situations of additional immunosuppression, titers of SARS-CoV-2 anti-spike IgG (−0.62 log_10_ BAU/mL; 95% CI: −1.01 to −0.23; P<0.01) and of SARS-CoV-2 surrogate NAB (−35.49%; 95% CI: −52.59 to −18.38; P<0.01) were significantly lower. Active tumor treatment was an independent factor for reduced immunogenicity and led to additionally significantly decreased SARS-CoV-2 anti-spike (−0.52 log_10_ BAU/mL; 95% CI: −0.84 to −0.20; P<0.01) and SARS-CoV-2 surrogate NAB titers (−17.88%; 95% CI: −30.59 to −5.16; P<0.01) (*Table 3* and *Figure 3*).

**Table 3:** Suspected factors influencing immunogenicity for SARS-CoV-2

	SARS-CoV-2 anti-spike IgG				SARS-CoV-2 surrogate NAB		
	Effect (log_10_ BAU/mL)	95% CI	P		Effect (%)	95% CI	P
Intercept (Ref.)	3.36	2.53 to 4.20	–		95.31	56.14 to 134.47	–
Under treatment	−0.52	−0.84 to −0.20	<0.01		−17.88	−30.59 to −5.16	<0.01
Additional immunosuppression^a^	−0.62	−1.01 to −0.23	<0.01		−35.49	−52.59 to −18.38	<0.01
Age	−0.01	−0.02 to 0.01	0.51		−0.12	−0.70 to 0.47	0.69
Sex (male)	−0.03	−0.30 to 0.24	0.84		−1.80	−14.04 to 10.44	0.77
History of SARS-CoV-2 infection	0.51	−0.17 to 1.18	0.15		10.80	−29.30 to 50.91	0.59
Time effect 12 weeks	−0.60	−0.82 to −0.38	<0.01		–	–	–
Treatment + time effect 12 weeks	0.25	−0.02 to 0.52	0.07		–	–	–

Shown are the results of multivariable linear mixed model analysis for the SARS-CoV-2 anti-spike IgG and multivariable linear model analysis for SARS-CoV-2 surrogate NAB. Results are reported by point estimates, 95% confidence intervals and associated P values. ^a^, co-medication with corticosteroids, methotrexate or calcineurin inhibitor, or HIV-infection. Ref., reference; IgG, immunoglobulin G; BAU, binding antibody units; NAB, neutralization antibody; CI, confidence interval; HIV, human immunodeficiency virus.

With increasing age and in male sex, there were minor, non-significant decreases in antibody titers. Infection with SARS-CoV-2 prior to vaccination led to a slight increase in SARS-CoV-2 anti-spike IgG as well as SARS-CoV-2 surrogate NAB (*Table 3*).

### Vaccination failure

Ineffective vaccination response rates were considered when levels of SARS-CoV-2 anti-spike IgG were <282.0 BAU/mL and/or of SARS-CoV-2 surrogate NAB <85.0% at week 12 after second vaccination.

Overall, 59.6% (N=31/52) of patients in the treatment group versus 29.4% (N=10/34) of patients in follow-up care failed to reach levels of SARS-CoV-2 anti-spike IgG ≥282.0 BAU/mL and of SARS-CoV-2 surrogate NAB ≥85.0% at week 12 after second vaccination (P=0.01). In our cohort, we discussed this constellation as total vaccination failure.

Ineffective levels of SARS-CoV-2 surrogate NAB (<85.0%) significantly differed between patients in the treatment group (67.3%; N=35/52) and patients in the control group (29.4%; N=10/34; P<0.01) 12 weeks after second vaccination. Concerning ineffective levels of SARS-CoV-2 anti-spike IgG (<282.0 BAU/mL), this difference was minor and non-significant at the same time-point: ineffective levels were found in 60.7% (N=34/56) in patients under active treatment versus in 40.0% (N=14/35) in patients in follow-up care (P=0.09). Isolated insufficient levels of SARS-CoV-2 surrogate NAB but sufficient levels of SARS-CoV-2 anti-spike IgG (N=4/86) or vice versa (N=4/86) were discussed as partial vaccination failure.

In view of the association between total and NABs with a coefficient of 0.93 (*Figure 2*), reaching titers of SARS-CoV-2 anti-spike IgG ≥482.0 BAU/mL (corresponding to 2.68 log_10_ BAU/mL), effective levels of SARS-CoV-2 surrogate NAB (≥85.0%) could be taken for granted probably indicating full protection from severe COVID-19 by vaccination in our cohort of patients (*Figure 2*). This could be found in 29.1% of patients in both groups (N=25/86).

### Effect of booster vaccination on SARS-CoV-2 anti-spike IgG and surrogate NABs

Finally, we assessed the effect of booster vaccination. Prior to booster vaccination, titers of SARS-CoV-2 anti-spike IgG further decreased in all patients (24 weeks after second vaccination) (*Figure 1*). Four weeks after booster vaccination, titers of SARS-CoV-2 anti-spike IgG significantly increased, both, in patients under active treatment (3.33 log_10_ BAU/mL; 95% CI: 3.10 to 3.56; P<0.01) and in patients in follow-up care (3.50 log_10_ BAU/mL; 95% CI: 3.22 to 3.79; P<0.01) compared to patients under active treatment and to patients in follow-up-care 12 weeks after second vaccination, respectively (*Figure 1*). Correspondingly, titers of SARS-CoV-2 surrogate NAB also increased in all patients (*Figure 2*). There was no significant difference between mean titers of patients under active treatment, independent of tumor type, and patients in follow-up care after booster vaccination (*Figure 1*). Effective titers of SARS-CoV-2 anti-spike IgG (≥282.0 BAU/mL) and of SARS-CoV-2 surrogate NAB (≥85%) were found in all patients. Patients with additional immunosuppression still showed significantly reduced titers of SARS-CoV-2 anti-spike IgG (−0.39 log_10_ BAU/mL; 95% CI: 0.18 to −2.22; P=0.03).

Titers of SARS-CoV-2 anti-spike IgG were again found to decrease 12 weeks after booster vaccination in patients under treatment (3.19 log_10_ BAU/mL; 95% CI: 2.91 to 3.46; P=0.55) as well as in patients in follow-up care (3.23 log_10_ BAU/mL; 95% CI: 2.88 to 3.58; P=0.63) compared to titers four weeks after booster vaccination.

Infections with SARS-CoV-2 (including B1.1.529.2) were found in three patients with active GI cancer and one patient in follow-up care.

### Adverse side effects

In self-report, injection-side pain (17.0%, N=21/125), erythema (6.0%, N=7/125) and swelling (6.0%, N=7/125) were the most common local side effects in all patients of our cohort. Regarding systemic side effects, fatigue (17.0%, N=21/125) was stated most frequently.

A case of grade IV pneumonitis after therapy with immune checkpoint inhibitor atezolizumab (programmed death-ligand 1 antibody) in a patient with HCC was discussed as possibly increased toxicity of anticancer therapy following SARS-CoV-2 vaccination. Moreover, a cutaneous reaction under panitumumab possibly related to the vaccination was documented in a patient with CRC.

## Discussion

In the present study, we showed that two vaccinations for SARS-CoV-2 in patients with active GI cancer under therapy was safe but induced significantly lower efficient response rates and higher rates of vaccination failure compared to patients in follow-up care being at least one year off any anticancer therapy. As impairment was particularly pronounced in patients with CRC, HCC and/or PBN, an association with the tumor type must be discussed. First booster vaccination improved antibody titers and balanced the differences between the groups. Since titers decreased again already 12 weeks after booster vaccination, earlier further booster vaccinations may be individually recommended in patients with GI cancer after antibody assessment.

No antibody titer associated with protection from severe COVID-19 was defined so far which would be decisive for recommendations concerning antiviral therapy, passive immunization and/or individual booster vaccinations. Titers of SARS-CoV-2 anti-spike IgG ≥264.0 BAU/mL were described as effective against B1.1.7 (alpha) previously (31). Since there was only one case of mild infection with SARS-CoV-2 in our cohort after second vaccination in patients in follow-up care, we could assume that mean titers of SARS-CoV-2 anti-spike IgG (≥282.0 BAU/mL) and of SARS-CoV-2 surrogate NAB (≥85.0%) were possibly effective to prevent severe COVID-19. This was also true after booster vaccination indicating even protection from B1.1.529.2 (i.e., omicron BA.2).

In our cohort of patients, we could demonstrate a stable association between titers of SARS-CoV-2 surrogate NAB and SARS-CoV-2 anti-spike IgG with a strong correlation coefficient of 0.93. Correlations in that range with values of up to 0.91 were described (32,33). By reaching a titer of SARS-CoV-2 anti-spike IgG ≥482.0 BAU/mL, protective levels of SARS-CoV-2 surrogate NAB (≥85.0%) could be assumed, indicating full protection by vaccination in our cohort.

Contrary to published studies so far, we analyzed a cohort of patients with active GI cancer and patients in follow-up care without current anticancer therapy. Unexpectedly, only 62.2% of patients with GI cancer under active anticancer treatment compared to 96.3% of patients in follow-up care (P<0.01) reached effective titers of SARS-CoV-2 anti-spike IgG (≥282.0 BAU/mL) four weeks after second vaccination in our cohort. Thus, we could identify active GI cancer and anticancer therapy as high-risk factors for reduced response to SARS-CoV-2 vaccination since patients in follow-up care achieved significantly higher titers for SARS-CoV-2 anti-spike IgG almost similar to that found in healthy people.

Consistent with several studies including patients with different kind of solid tumors, mean antibody titers of SARS-CoV-2 anti-spike IgG were also significantly lower in the treatment group. However, the proportion of patients with solid cancer with positive antibody responses was comparatively higher than in our cohort and ranged from 84.1% to 95.0% of cases versus 100% in participants of healthy control groups (10-12,14-16). Interestingly, our patients seemed to resemble more patients with hematological cancer who also showed low positive response rates of only 60.0% after two vaccinations than patients with solid tumors (13,15).

Similar to our findings, titers decreased overtime which we could be proved in both groups of patients highlighting the necessity of booster vaccination. However, we observed a more pronounced decreasing in the group of patients with active GI cancer under treatment. Other cohorts including all solid cancer still had positive response rates of 79.0% compared to 84.0% in healthy participants (P=0.32) in a follow up of 6 months after second vaccination (34).

Regarding levels of surrogate NAB, we found a more pronounced impairment in patients with active GI cancer compared to patients in follow-up care. Twelve weeks after second vaccination, only 32.7% of patients under treatment versus 70.6% of patients in follow-up care reached protective levels of SARS-CoV-2 surrogate NAB (≥85.0%), empathizing again a clinically relevant impairment in patients with active GI cancer. Data of SARS-CoV-2 surrogate NAB in patients with cancer are rare and therefore difficult to compare. Rates of positive levels of SARS-CoV-2 surrogate NAB ranged from 81.3% to 69.0% in patients with solid cancer without distinguishing between tumor types and without a definition concerning protective levels (12,21). Interestingly, patients with hematological malignancies showed worse rates, down to 41.0% in patients with chronic lymphatic leukemia (CLL) or B-cell-non-Hodgkin-lymphoma (B-NHL), which was similar to our findings (17,19).

Finally, rates of total vaccination failure (SARS-CoV-2 anti-spike IgG <282.0 BAU/mL and SARS-CoV-2 surrogate NAB <85.0%) were significantly higher in patients with GI cancer under active treatment (59.6%) than in follow-up patients with a past medical history of GI cancer (29.4%). Studies focusing only on SARS-CoV-2 anti-spike IgG found positive levels between 81.0% and 95.5% in patients with solid tumors (11,13). Patients with CLL or B-NHL had even lower rates with only 52% and 49%, respectively (17-19). Comparing hematological and solid cancers, vaccination response rates differed significantly (13). Again, our cohort with only GI-cancer seemed to resemble patients with hematological malignancies, more than patients with other solid tumors.

Partial vaccination failure (isolated insufficient levels of SARS-CoV-2 surrogate NAB but sufficient levels of SARS-CoV-2 anti-spike IgG or vice versa) was rare in our cohort but must also be taken into consideration indicating a considerable discrepancy in real effectiveness of vaccination. Although the exact form of association is yet unclear, both, SARS-CoV-2 anti-spike IgG as well as SARS-CoV-2 surrogate NAB are crucial for protection from infection or severe COVID-19 (22,35-38).

In order to understand, why our cohort of patients with active GI cancer showed unexpectedly highly impaired immunogenicity to vaccination for SARS-CoV-2 compared to data in patients with solid tumors in general published to date, we further analyzed our patients regarding tumor entity, therapy and further risk factors. Interestingly, our cohort of patients included a comparatively high proportion of patients with primary pancreatico-hepato-biliary tumors: 23.5% of cases were HCC and 24.5% PBN, followed by 20% CRC. Studies proving impaired immunogenicity after vaccination for SARS-CoV-2 in patients with solid cancer only included up to 25% patients suffering from GI cancer (11,12). Moreover, for the time being, no differentiated analyses regarding different GI tumor types separately were performed in these studies so far. However, the differentiated analysis presented here evidently explained our results, since SARS-CoV-2 anti-spike IgG were lowest in CRC and SARS-CoV-2 surrogate NAB were lowest in patients with HCC and PBN. Especially, hepato-pancreato-biliary tumors as well as secondary hepatic metastases from GI cancers are well known to impair host immunity, somewhat explaining the results concerning impaired SARS-CoV-2 immunogenicity (24,25). Furthermore, underlying liver cirrhosis/ fibrosis especially in patients with HCC and in part with CCC is linked to immunodeficiency (26). In general, the capacity to develop adequate response rates to vaccinations in patients with liver dysfunction is reduced as shown for hepatitis B and/ or pneumococcal vaccines previously (27,28). Finally, an under-release of cytokines must be taken into account in patients with CRC as shown previously (39).

Regarding the effects of anticancer therapy, we found that patients under immune checkpoint inhibition seemed to form higher SARS-CoV-2 surrogate NAB titers compared to patients under cytostatic chemotherapy as shown previously (12). Compared to therapy regimes of other solid tumor types (for instance breast or lung cancer), use of immune checkpoint inhibitors is less common in patients with GI cancer also partly explaining the more pronounced impairment of SARS-CoV-2 immunogenicity in patients with GI cancer.

Considering the high proportion of patients with HCC, PBN and CRC under anticancer and the reduced use of immunotherapy, impaired COVID-19 immunogenicity in patients with active GI cancer seems obviously to be tumor-related. Of note, no impact of the disease status (local, metastatic, recurrent) on immune responses was observed. Regarding risk for impaired COVID-19 immunogenicity, GI tumor types could be classified as a solid borderline tumor group with similarities to hematological malignancies most likely explained by an underlying immunodeficiency. Further studies in other cohort of patients to confirm these findings and further particularities of these patients should be studied next.

Furthermore, additional immunosuppression was found to worsen SARS-CoV-2 immunogenicity with significantly lower titers of SARS-CoV-2 anti-spike IgG and SARS-CoV-2 surrogate NAB. This reduction was more evident in patients under active tumor treatment and was in line with other studies in cancer patients (10,12,14,16). Contrary, infection with SARS-CoV-2 prior to vaccination appeared to increase immunogenicity after SARS-CoV-2 vaccination since these patients showed similar increase in total and in surrogate NAB compared to patients in follow-up care. Other studies also demonstrated a favorable effect of a past medical history of COVID-19 on antibody titers in healthy persons as well as in patients with cancer (12,40,41).

Regarding age and sex, no significant influence on antibody titers could be observed. Data on the impact of age and sex are inconsistent as some showed no evidence for an effect (10), while others demonstrated a better immune response in female patients and patients under the age of 65 (12,17).

According to our data, booster vaccination can overcome differences between patients under active treatment and in follow-up care, since all patients reached effective levels of SARS-CoV-2 anti-spike IgG (≥282.0 BAU/mL) and NAB (≥85.0%). This is in line with clinical observations that rates of confirmed COVID-19 or severe COVID-19 were significantly reduced after booster vaccination (42). Moreover, the immunogenic potential of booster vaccination could be confirmed, even for patients with cancer having been seronegative after first and second vaccination (43,44). In patients with solid cancer, NAB against omicron was detectable in 90% of patients after booster vaccination (45). Especially for patients with hepatobiliary carcinoma, booster vaccinations are thus already recommended (20). Of note, data on the effect of booster vaccination in patients with cancer are sparse for the time being. However, as we found that titers of SARS-CoV-2 anti-spike IgG again decreased 12 weeks after booster vaccination, earlier further booster vaccinations should be taken into consideration individually in patients with GI cancer after antibody assessment.

The present study has some limitations. Consequent follow-up of all patients was not possible as some patients died, and some patients had a reduced performance status while continuing their medical treatment in the department of palliative care. While presenting extensive data on humoral immunogenicity for SARS-CoV-2, data on cellular immune response are missing.

The strengths of our study is a more conservative analysis than previous studies since we not only regarded positive response rates to vaccination but defined titers being linked to protection. However, this makes it difficult to compare our data with other studies due to different cut-off values for antibody testing (sole test positivity versus reaching individually defined effective titers), different time points for antibody testing and heterogeneous distribution of tumor entities in other studies. This might have contributed to the overall worse response rates in patients with GI-cancer under active treatment compared to patients with other solid tumors in the same situation.

## Conclusions

In summary, we showed that patients with GI cancer under anticancer therapy reached unexpectedly worse response rates to vaccination for SARS-CoV-2 than comparable patients in follow-up care. Impairment was particularly pronounced for patients with HCC, PBN and CRC. Thus, we claim that effects are tumor-related due to underlying immunodeficiency. However, we could define levels of SARS-CoV-2 anti-spike IgG (≥282.0 BAU/mL) as well as of SARS-CoV-2 surrogate NAB (≥85.0%) having been associated with protection from severe COVID-19 marking a landmark for clinicians as well as for future studies. Booster vaccination finally led to effective antibody titers in all tested patients. Considering waning immunity as well as antibody escape phenomena of variant of concern Omicron (46,47), follow-up analyses putting emphasis on long-term immunogenicity following vaccination with Omicron-adapted vaccines into account.

## Supplementary

The article’s supplementary files as

10.21037/jgo-22-1065 10.21037/jgo-22-1065 10.21037/jgo-22-1065

## Notes

*Ethical Statement:* The authors are accountable for all aspects of the work in ensuring that questions related to the accuracy or integrity of any part of the work are appropriately investigated and resolved. The study was performed in accordance with the Declaration of Helsinki (as revised in 2013) and approved by the institutional review board of the Medical Faculty of the University of Bonn (No. 341/17 and 023/22). Written informed consent was obtained from all participating patients.
