# Supplementary material for: Impaired immunogenicity after vaccination for SARS-CoV-2 in patients with gastrointestinal cancer: does tumor entity matter?
Source: J Gastrointest Oncol. 2023 Jun 26;14(3):1218–34. doi: 10.21037/jgo-22-1065 (PMC10331752; doi:10.21037/jgo-22-1065)
Supplement: Supplementary file 2 [file jgo-14-03-1218-dss.pdf]

Data Sharing Statement

**Article Info**	https://dx.doi.org/10.21037/jgo-22-1065	
**Item**	**Question**	**Authors' Response**<br>**（place "-" if not applicable）**
1	Would you like to share data collected for your study to others?	Yes, we will share all data anonymously on request.
2	If not, would you like to share the reason for your decision?	-
3	What data in particular will be shared?	We will share all raw and patient data anonymously on request.
4	Any other documents will be shared? Such as study protocol, statistical analysis plan, informed consent form, clinical study report, analytic code.	All the mentioned documents could be shared on request.
5	When will data availability begin?	On request, all data are available from now on.
6	When will data availability end?	Data will be available for at least 10 years.
7	To whom will you share the data?	On request, data will be shared to others. Each decision will be individualized.
8	For what type of analysis or purpose?	Predominantly for cooperation.
9	How or where can the data/documents be obtained?	Data can be obtained by personally contacting the corresponding author.
10	Any other restrictions?	No.
