# Supplementary material for: Impaired immunogenicity after vaccination for SARS-CoV-2 in patients with gastrointestinal cancer: does tumor entity matter?
Source: J Gastrointest Oncol. 2023 Jun 26;14(3):1218–34. doi: 10.21037/jgo-22-1065 (PMC10331752; doi:10.21037/jgo-22-1065)
Supplement: Supplementary file 1 [file jgo-14-03-1218-rc.pdf]

STROBE Statement—checklist of items that should be included in reports of observational studies

Section/item	Item No	Recommendation	Reported on Page Number/Line Number	Reported on Section/Paragraph
**Title and abstract**	1	(a) Indicate the study's design with a commonly used term in the title or the abstract	3/ 55 - 57	Abstract-Methods
		(b) Provide in the abstract an informative and balanced summary of what was done and what was found	3/ 53 - 73	Abstract
**Introduction**				
Background/ rationale	2	Explain the scientific background and rationale for the investigation being reported	4 - 5/ 78 - 105	Introduction
Objectives	3	State specific objectives, including any prespecified hypotheses	5/ 109 - 123	Study design
**Methods**				
Study design	4	Present key elements of study design early in the paper	5/ 109 - 123	Study design
Setting	5	Describe the setting, locations, and relevant dates, including periods of recruitment, exposure, follow-up, and data collection	5 - 6/ 109 - 145	Study design and Patient characteristics ...
Participants	6	(a) ***Cohort study***—Give the eligibility criteria, and the sources and methods of selection of participants. Describe methods of follow-up<br>***Case-control study***—Give the eligibility criteria, and the sources and methods of case ascertainment and control selection. Give the rationale for the choice of cases and controls<br>***Cross-sectional study***—Give the eligibility criteria, and the sources and methods of selection of participants	5 - 6/ 125 - 145	Patient characteristic ...
		(b) ***Cohort study***—For matched studies, give matching criteria and number of exposed and unexposed<br>***Case-control study***—For matched studies, give matching criteria and the number of controls per case	5 - 6/ 125 - 145	Patient characteristic ...
Variables	7	Clearly define all outcomes, exposures, predictors, potential confounders, and effect modifiers. Give diagnostic criteria, if applicable	5/ 108 - 123	Study Design
Data sources/ measurement	8*	For each variable of interest, give sources of data and details of methods of assessment (measurement). Describe comparability of assessment methods if there is more than one group	6 - 7/ 147 - 167	Antibody assessment
Bias	9	Describe any efforts to address potential sources of bias	19/ 459 - 470	Discussion: Limitations
Study size	10	Explain how the study size was arrived at	5-6/ 124 - 145	Patients characteristics...
Quantitative variables	11	Explain how quantitative variables were handled in the analyses. If applicable, describe which groupings were chosen and why	7-8/ 169 - 187	Statistical analysis

Statistical methods	12	(a) Describe all statistical methods, including those used to control for confounding	7 - 8/ 170 - 187	Statistical analysis
		(b) Describe any methods used to examine subgroups and interactions	7 - 8/ 170 - 187	Statistical analysis
		(c) Explain how missing data were addressed	19/ 459 - 470	Discussion: Limitations
		(d) ***Cohort study***—If applicable, explain how loss to follow-up was addressed<br>***Case-control study***—If applicable, explain how matching of cases and controls was addressed<br>***Cross-sectional study***—If applicable, describe analytical methods taking account of sampling strategy	19/ 459 - 470	Discussion: Limitations
		(e) Describe any sensitivity analyses	7 - 8/ 170 - 187	Statistical analysis
**Results**				
Participants	13*	(a) Report numbers of individuals at each stage of study—eg numbers potentially eligible, examined for eligibility, confirmed eligible, included in the study, completing follow-up, and analysed	8/ 191 - 192<br>10/ 229	Patient baseline characteristics, Table 2
		(b) Give reasons for non-participation at each stage	19/ 459 - 470	Discussion: Limitations
		(c) Consider use of a flow diagram	NA	NA
Descriptive data	14*	(a) Give characteristics of study participants (eg demographic, clinical, social) and information on exposures and potential confounders	8/ 191 - 197 + Table 1	Patient baseline characteristics + Table 1
		(b) Indicate number of participants with missing data for each variable of interest	8/ 199 (Table 1)	Table 1
		(c) ***Cohort study***—Summarise follow-up time (eg, average and total amount)	5/ 109 - 120	Study design
Outcome data	15*	***Cohort study***—Report numbers of outcome events or summary measures over time	10/229	Table 2
		Case-control study—Report numbers in each exposure category, or summary measures of exposure	NA	NA
		Cross-sectional study—Report numbers of outcome events or summary measures	NA	NA
Main results	16	(a) Give unadjusted estimates and, if applicable, confounder-adjusted estimates and their precision (eg, 95% confidence interval). Make clear which confounders were adjusted for and why they were included	10/ 229<br>11/ 262	Table 2<br>Table 3
		(b) Report category boundaries when continuous variables were categorized	NA	NA
		(c) If relevant, consider translating estimates of relative risk into absolute risk for a meaningful time period	NA	NA
Other analyses	17	Report other analyses done—eg analyses of subgroups and interactions, and sensitivity analyses	10 - 13/ 247 - 319	Impact of ....
**Discussion**				
Key results	18	Summarise key results with reference to study objectives	13/ 21-30	Discussion
Limitations	19	Discuss limitations of the study, taking into account sources of potential bias or imprecision. Discuss both direction and magnitude of any potential bias	19/ 459 - 470	Discussion

Interpretation	20	Give a cautious overall interpretation of results considering objectives, limitations, multiplicity of analyses, results from similar studies, and other relevant evidence	19/ 472 - 482	Discussion
Generalisability	21	Discuss the generalisability (external validity) of the study results	13 - 19/ 321 - 482	Discussion
**Other information**				
Funding	22	Give the source of funding and the role of the funders for the present study and, if applicable, for the original study on which the present article is based	20/ 490	Source of funding

*Give information separately for cases and controls in case-control studies and, if applicable, for exposed and unexposed groups in cohort and cross-sectional studies.

Note: An Explanation and Elaboration article discusses each checklist item and gives methodological background and published examples of transparent reporting. The STROBE checklist is best used in conjunction with this article (freely available on the Web sites of PLoS Medicine at http://www.plosmedicine.org/, Annals of Internal Medicine at http://www.annals.org/, and Epidemiology at http://www.epidem.com/). Information on the STROBE Initiative is available at www.strobe-statement.org.

Article Information: https://dx.doi.org/10.21037/jgo-22-1065
*As the checklist was provided upon initial submission, the page number/line number reported may be changed due to copyediting and may not be referable in the published version. In this case, the section/paragraph may be used as an alternative reference.
